# Excision of the Tongue

**Published:** 1866-04

**Authors:** James Syme

**Affiliations:** Ruteland Street, Edinburgh


ART. III.---*Excision of the Tongue.*---By JAMES SYME, F. R. S. E., etc.

About twelve months ago I communicated a case in which the tongue had been completely removed by excision, on account of extensive disease that threatened to prove fatal by preventing the admission of nourishment. This account was necessarily limited to the operation and its immediate effects, as sufficient time had not elapsed for determining whether or not the relief afforded would prove permanent, or how far the powers of deglutition, articulation, and taste would be restored. After his return home to Manchester, the patient sent me favorable reports of his progress, but certainly not such as to convey an adequate idea of the improvement that had taken place since he came under my care. He was then emaciated and bent down by long-continued suffering, unable to articulate, so as to require a slate and pencil for expressing his wishes, and swallowing even fluids with such extreme difficulty as to feel on the point of starvation. My surprise may, therefore, be imagined when on the 10th of September last he unexpectedly made his appearance, erect and vigorous, and, seeing that I did not recognize him, announced his name in a loud, clear voice. The feeling thus excited was not lessened by learning that while traveling in the Highlands he had dined at a table d'hotes, and entered into conversation without betraying the deficiency under which he laboured. Very much astonished by a result so much better than could have been anticipated, I requested a number of my medical friends to join me in examining the state of matters. Professor Goodsir and Mr. Nassmyth having satisfied themselves that no vestige of the tongue remained, various observations were made with regard to articulation and other functions of the absent organ; and Mr. Annadale afterward instituted a more particular inquiry, of which he has given me the following report:

"The lips and jaw-bone, where divided, were soundly united without any deformity. The opening between the mouth and pharynx was much diminished in size and irregular in shape from contraction of the fauces and soft palates, which were drawn downward and forward more to the right than to the left side, from the mucous membrane at that part having participated in the disease and been removed along with the tongue. Mr. W. says that he can swallow as well as ever, provided that the food is finely divided or fluid. He is also able to masticate solid substances, although difficulty is sometimes experienced from their getting into awkward parts of the mouth. In ordinary speech his words are wonderfully clear and distinct, and he can sing without difficulty. All the vowels and words composed of them are articulated perfectly, and also the following consonants: B, C, F, H, K, L, M, N, P, Q, R, V, W. D is pronounced "dthe," J "the," G like "sjee," S is a lisp. His taste is impaired, but still enables him to distinguish different articles and their respective qualities, as grouse from partridge, bitters from sweets, good beer from bad beer, etc. He has remarked that the seat of sensation lies somewhere in the throat, since there is no recognition of taste previous to the act of swallowing; and, in order to ascertain the truth of this point more precisely, the following experiments were made:

"1. A strong solution of salt was applied, by means of a camel-hair brush, to the fauces, palate, floor of the mouth, lips, and inner surface of the cheek, with the result of something being felt in the mouth, but no idea formed as to its nature.

"2. About a quarter of a teaspoonful of finely powdered sugar was placed on the floor of the mouth, and having been allowed to remain there a few seconds, was then brought thoroughly into contact with every part of the cavity without any recognition of its nature; but when a little water was added and swallowed, the taste was immediately perceived.

"3. The same experiment was repeated with another substance (salt), and with the same result."

It has long been known that large portions of the tongue may be removed without destroying or materially impairing the power of articulation, but I am not aware of any case on record in which it has remained so perfect after complete removal of the organ. Of the facts above mentioned, the one that seems most curious is the connection between taste and deglutition; from which it appears that the latter is essential for the full perception of the former. If the pleasure of taste could be perfectly gratified by mastication without deglutition, there would be no limit to the consumption of food; but the instinctive desire to swallow an agreeable morsel affords a check to any such abuse.

As the nature of the disease was not particularly described in relating the operation, the microscopic structure exhibited by the tumor (for which I am indebted to Mr. Annadale), may be given to show that it possessed the character of epithelial cancer.

[*London Lancet*, Jan. 27.

Ruteland Street, Edinburgh, January, 1866.

---

In the "London Lancet," February 3rd, is a communication from Dr. Wm. McCormack, of Belfast, drawn out by the above report of Professor Syme's case. He thus remarks: "During the winter of 1857, I remember seeing in the reception hall of the Imperial Academy of Medicine, a man from whom M. Ricord had excised the entire tongue some time previously. I looked into his mouth, and saw that there was not a remnant of the tongue left. He was of middle age, well nourished, and contented-looking. I was inexpressibly surprised upon hearing this man talk fluently and with perfect ease, and as far as I could detect, without any deficiency whatever in the power of articulation." G. C. B.

[*Cincinnati Journal of Medicine.*

